# Mapping land cover on Reunion Island in 2017 using satellite imagery and geospatial ground data

**DOI:** 10.1016/j.dib.2019.104934

**Published:** 2019-12-05

**Authors:** Stéphane Dupuy, Raffaele Gaetano, Lionel Le Mézo

**Affiliations:** a CIRAD, UMR TETIS, F-97410 Saint-Pierre, Réunion, France; b CIRAD, UMR TETIS, F-34398 Montpellier, France; c TETIS, Univ Montpellier, AgroParisTech, CIRAD, CNRS, IRSTEA, Montpellier, France; d CIRAD, UPR AIDA, F-97410 Saint-Pierre, Réunion, France

**Keywords:** Remote sensing, Land cover map, Spatial database, Landsat-8, Sentinel-2, Pleiades

## Abstract

We here present a reference database and three land use maps produced in 2017 over the Reunion island using a machine learning based methodology. These maps are the result of a satellite image analysis performed using the Moringa land cover processing chain developed in our laboratory. The input dataset for map production consists of a single very high spatial resolution Pleiades images, a time series of Sentinel-2 and Landsat-8 images, a Digital Terrain Model (DTM) and the aforementioned reference database. The Moringa chain adopts an object based approach: the Pleiades image provides spatial accuracy with the delineation of land samples via a segmentation process, the time series provides information on landscape and vegetation dynamics, the DTM provides information on topography and the reference database provides annotated samples (6256 polygons) for the supervised classification process and the validation of the results. The three land use maps follow a hierarchical nomenclature ranging from 4 classes for the least detailed level to 34 classes for the most detailed one. The validation of these maps shows a good quality of the results with overall accuracy rates ranging from 86% to 97%. The maps are freely accessible and used by researchers, land managers (State services and local authorities) and also private companies.

**Table:** Specifications Table

Subject	Computer Science, Earth Sciences, Social Sciences
Specific subject area	Remote Sensing, GIS, Land Cover Map
Type of data	Vector
How data were acquired	The reference database was created with the QGIS software (www.qgis.org) For the production of land use maps, the Moringa processing chain uses the Orfeo ToolBox software (www.orfeo-toolbox.org) with scripts written in Python language. The source code of the Moringa processing chain is available at https://gitlab.irstea.fr/raffaele.gaetano/moringa.git
Data format	Raw data (Shapefile, Esri)
Parameters for data collection	For the construction of the reference database, the objective of the choice of plots was (i) a good representativeness of the class and (ii) a homogeneous distribution over the territory
Description of data collection	To create the reference database, we used existing IGN databases but also field surveys acquired with a GPS and the knowledge of professionals specialized in their field. For the land use maps, we used a supervised classification method of satellite images (Sentinel2, Landsat8 and Pleiades) based on the Random Forest algorithm driven by the reference database mentioned above. We produced the three land use maps using the reference database and satellite image classifications as described below.
Data source location	Réunion Island is a French oversea region located in the Indian Ocean near Mauritius and Madagascar (upper left corner: 20°50′30.81″S and 55°12′51.92″E//lower right corner: 21°27′02.43″S and 55°48′51.90″E)
Data accessibility	Repository name: CIRAD Dataverse Data identification number: - Land use map: Dupuy, Stéphane; Gaetano, Raffaele, 2019, “Reunion island - 2017, Land cover map (Pleiades)”, doi:10.18167/DVN1/RTAEHK, CIRAD Dataverse, V2 - Reference database: Dupuy, Stéphane, 2019, “Reunion Island - 2017, reference spatial database”, doi:10.18167/DVN1/TOARDN, CIRAD Dataverse, V3 Direct URL to data: Data are referenced in the CIRAD Dataverse and are hosted on CIRAD's Aware Geographic Catalog. The web links are in the following files. - Land use map: http://dx.doi.org/10.18167/DVN1/RTAEHK - Learning database: http://dx.doi.org/10.18167/DVN1/TOARDN
Related research article	**Gaetano R., Dupuy S., Lebourgeois V., Le Maire G., Tran A., Jolivot A., Bégué A.** 2019. The MORINGA Processing Chain: Automatic Object-based Land Cover Classification of Tropical Agrosystems using Multi-Sensor Satellite Imagery, in ESA Living Planet Symposium (LPS 2019), Milan, Italy.

**Table:** 

**Value of the Data** - the referenced land cover maps provide an unprecedented overview of the entire territory of Reunion Island, with a significant potential impact in various tasks related to land, agriculture and environmental monitoring. - These maps can be used in GIS to monitor changes in the territory and help managers make decisions about urbanization, natural and agricultural land management. - The reference database can be used by remote sensing specialists to assess new methods for land cover mapping and other classification algorithms. - All data provided is georeferenced and in vector format for use in GIS tools in future projects.

## 1 Data

**Fig. 1:**
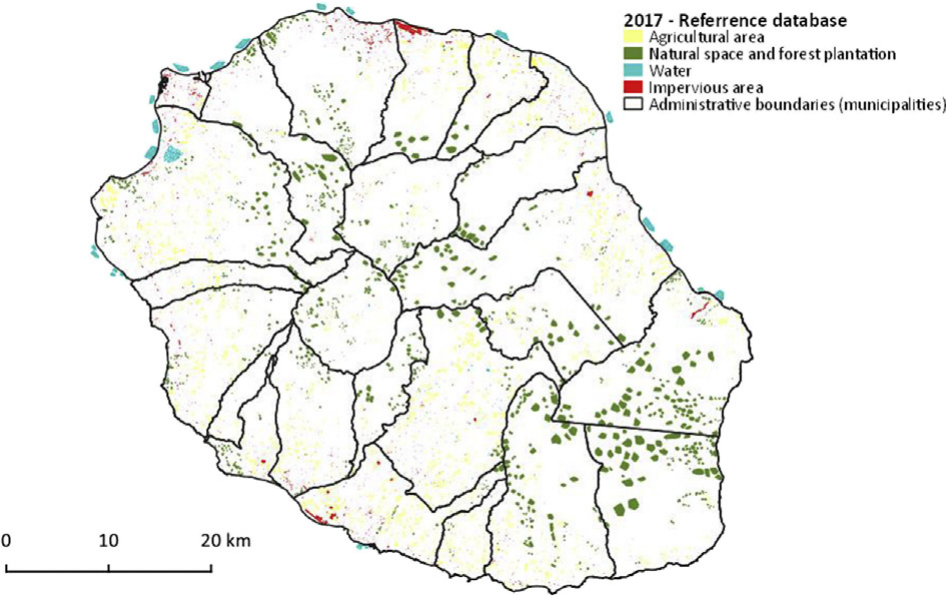
Distribution of polygons in the first level. Vector file in ESRI shape format available here: Dupuy, Stéphane, 2019, “Reunion Island - 2017, reference spatial database”, https://doi.org/10.18167/DVN1/TOARDN, CIRAD Dataverse, V3.
• Three land use maps produced starting from a dataset including a Very High Spatial Resolution (VHRS) Pléiades image, a time series of HRS Sentinel-2 and Landsat-8 images and a digital terrain model. These maps correspond to three levels of land use nomenclature (from 4 to 34 classes) and are distributed in vector format (shapefile). Each geometry corresponds to an object provided by the segmentation of the Pleiades image, attributed using reflectances, radiometric and textural indices computed on the different remote sensing images available plus topographic information (altitude and slope). Such objects are individually classified using a supervised classification algorithm trained using the reference database described above. The validation of the maps gives overall accuracies ranging from 86% for the most detailed level to 98% for the least detailed level. These maps were produced as part of the GABIR project, for which we needed precise information on crop location. The nomenclature is therefore detailed on this type of land use but we have also detailed the natural areas so that these maps can be used in different themes. The three maps produced are illustrated in Fig. 2, Fig. 3, Fig. 4.

The data described in this data paper are of two different types related to land use on Reunion Island for the year 2017:

• A reference database consisting of GIS vector dataset in ESRI shapefile format composed of 6256 polygons representative of the diversity of land use on Reunion Island. This data is used in a supervised classification process to train an algorithm to recognize land use classes on a set of variables from high and very high spatial resolution satellite images. A part of the polygons (20%) is dedicated to the validation of classification results (Cf. Fig. 1);

**Fig. 2:**
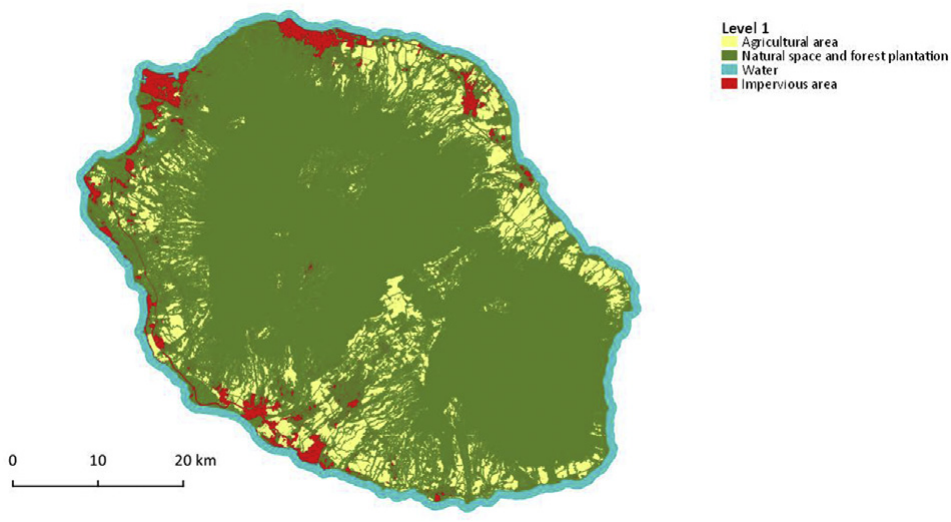
Map of land cover groups with 4 classes, corresponds to Level 1. Vector file in ESRI shape format available here: Dupuy, Stéphane; Gaetano, Raffaele, 2019, “Reunion island - 2017, Land cover map (Pleiades)”, https://doi.org/10.18167/DVN1/RTAEHK, CIRAD Dataverse, V2.

**Fig. 3:**
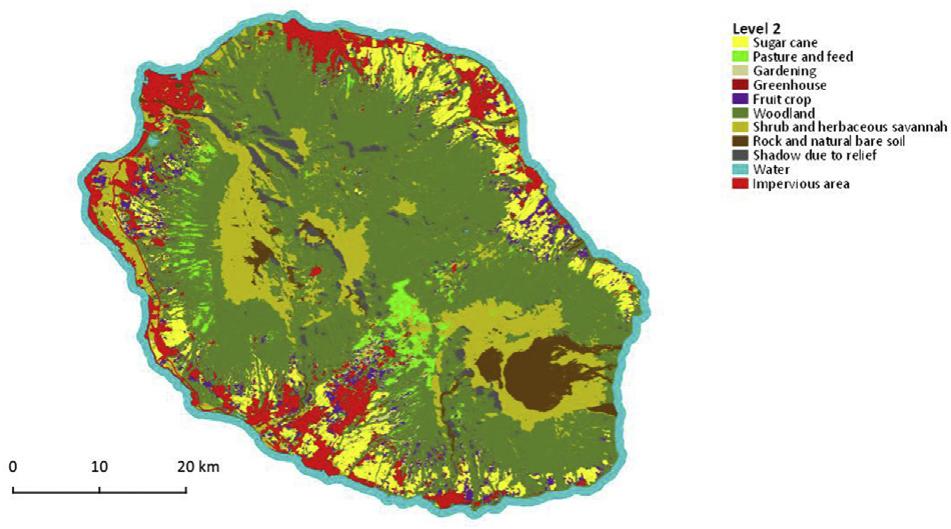
Map of crop group with 11 classes, corresponds to Level 2. Vector file in ESRI shape format available here: Dupuy, Stéphane; Gaetano, Raffaele, 2019, “Reunion island - 2017, Land cover map (Pleiades)”, https://doi.org/10.18167/DVN1/RTAEHK, CIRAD Dataverse, V2.

**Fig. 4:**
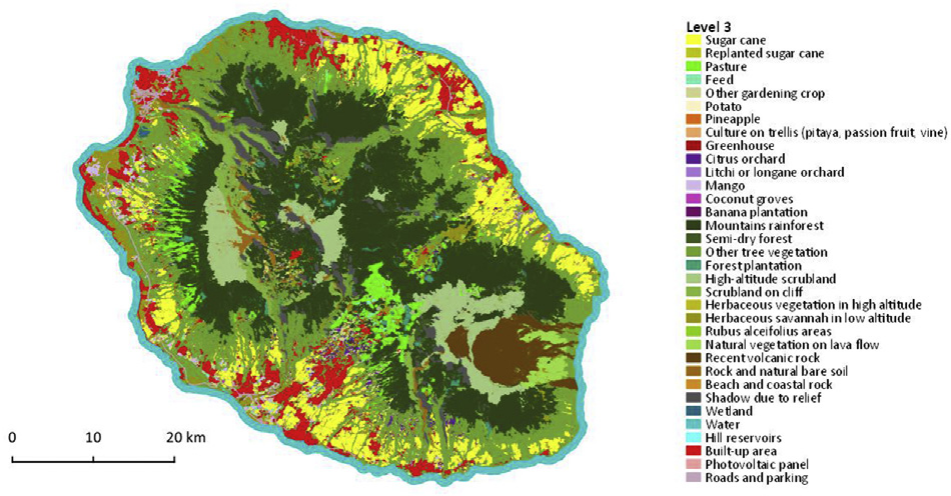
Map of crop type with 34 classes, corresponds to Level 3. Vector file in ESRI shape format available here: Dupuy, Stéphane; Gaetano, Raffaele, 2019, “Reunion island - 2017, Land cover map (Pleiades)”, https://doi.org/10.18167/DVN1/RTAEHK, CIRAD Dataverse, V2.

Final maps in ESRI shapefile format are delivered in the local UTM projection (WGS 84 UTM 40 South, EPSG code 32740).

Data are referenced in the CIRAD Dataverse and are hosted on CIRAD's Aware Geographic Catalog (http://aware.cirad.fr). The advantage of distributing geographical data with this system is that it is possible to simply visualize them, to use them directly on GIS software with web services (WFS: Web Feature Service or WMS: Web Map Service) or simply download them.

## 2 Experimental design, materials, and methods

### 2.1 Materials

#### 2.1.1 Reference database and nomenclature

The reference database is organized according to a multi-level nomenclature (Cf. Table 1). For class representativeness purposes it is important that the number of polygons in each class meets the minimum requirement of 60 entities at the most detailed level of the nomenclature.

**Table 1:** Nomenclature presenting the three levels of precision and the number of polygons in each class used for learning and validation.

Level 1	Level 2	Level 3	Number of polygons
Agricultural area	Sugar cane	Sugar cane	779
		Replanted sugar cane	79
	Pasture and feed	Pasture	264
		Feed	318
	Gardening	Other gardening crop	353
		Potato	83
		Pineapple	281
		Culture on trellis (pitaya, passion fruit, vine)	43
	Greenhouse	Greenhouse	260
	Fruit crop	Citrus orchard	318
		Litchi or longane orchard	75
		Mango	156
		Coconut groves	44
		Banana plantation	174
Natural space and forest plantation	Woodland	Mountains rainforest	134
		Semi-dry forest	42
		Other tree vegetation	248
		Forest plantation	146
	Shrub and herbaceous savannah	High-altitude scrubland	81
		Scrubland on cliff	74
		Herbaceous vegetation in high altitude	50
		Herbaceous savannah in low altitude	124
		Rubus alceifolius areas	113
		Natural vegetation on lava flow	64
	Rock and natural bare soil	Recent volcanic rock	72
		Rock and natural bare soil	182
		Beach and coastal rock	45
	Shadow due to relief	Shadow due to relief	81
Water	Water	Wetland	61
		Water	43
		Hill reservoirs	73
Impervious area	Impervious area	Built-up area	1164
		Photovoltaic panel	65
		Roads and parking	167
		Total	6256

To build this database we use data disseminated by the French Institute of Geography (IGN) and available on condition of eligibility. These are the products: RGE Alti®, “BD Topo®” and “RPG”. They are available on this website: http://professionnels.ign.fr.

The reference database for 2017 consists of 6256 plots. We will give a brief description of the sources and techniques used to build it according to the various land use groups:

• For agricultural areas, we have information on certain cultivated plots. These are the declarations made by farmers to apply for EU subsidies: the *Registre Parcellaire Graphique* (RPG, the French Land Parcel Identification System). This data is disseminated in France by the French Institute for Geographical and Forestry Information (IGN). The description of this data is available here (in French): http://professionnels.ign.fr/doc/DC_DL_RPG-2-0.pdf. These vector data are of good quality and can be used as a model to locate crops. The release times imply that we use the RPG for year N −1. It is therefore necessary to check the correct consistency of the data by photo-interpretation of the VHSR image. The RPG provides little information on tree crops. Therefore, for these classes we have called on colleagues specialised in mango, lychee and citrus fruit cultivation who are familiar with their area and can locate plots in the VHSR image. Field surveys were conducted using GPS for market gardening crops. The plots of the “greenhouse or shade cultivation” class are derived from the “industrial building” layer of the IGN's “BD TOPO” product, and selected among different height levels to keep a sufficient diversity of greenhouse types. Each of the polygons was verified by photo-interpretation using the Pleiades image. If the greenhouse or shade was not visible in the image, the polygon was deleted.
• For natural areas, there is no regularly updated mapping, but the main classes can be recognized from the GIS layers of the State services that manage these areas (ONF: National Forestry Office and DEAL: Regional Office of the Ministry of Environment, Development and Housing). The validity date of this data is not known. We therefore checked the coherence of each selected polygon against the VHSR image. Two specific classes have been added (identified by photo-interpretation) to address the problems of satellite images: a class of shadows due to the island's steep terrain (areas which are never visible because of shades) and a class of vegetation located on steep slopes facing the morning sun called “rampart moor”.
• For water and wet spaces, the “marsh”, “water” and “hillside retention” classes were obtained by photo-interpretation of the 2017 Pleiades image. These classes are easily recognizable on this type of image.
• For urban spaces, we randomly selected polygons from the IGN's “BD TOPO” product. For the housing type building, four building height classes have previously been created (depending on the height of the layer field) in order to preserve a good diversity of the types of buildings present on the island. A random selection of polygons from each class was then made. The “built” layer was completed by a random selection of industrial buildings from the “industrial building” layer of the IGN's “BD TOPO” product. This selection was made in the “nature” field of the layer (i.e. the following types: silo, industrial and livestock). These different polygons were finally merged into a single built up class grouping all types of buildings while maintaining a good representativeness. The “photovoltaic panel” class was obtained by photo-interpretation of the polygons on the 2017 Pleiades image

The contours of each polygon have been checked to ensure that they are perfectly overlapping on the THRS image used for the classification process and slightly eroded (negative buffer) in order to limit edge effects in the training process.

The reference database was used in the works described in Refs. [1,2].

#### 2.1.2 Images

➢ Very High Spatial Resolution (VHSR):

We use VHSR images acquired in the framework of the Kalideos project, driven by CNES (*Centre National d’Etudes Spatiales*: government agency responsible for shaping and implementing France's space policy in Europe). Pleiades images are not free and are available under the condition of eligibility via the Kalideos project website (https://www.kalideos.fr). In 2017, we dispose of 15 Pleiades scenes covering the whole island. Several zones are acquired multiple times because of cloud coverage. Pre-processed images are provided: radiometric correction to *Top-Of-Atmosphere* (TOA) level, precise orthorectification using high quality IGN's Pléiades database as a spatial reference (see Fig. 7, image mosaic).

➢ High Spatial Resolution (HSR):

The Sentinel 2A and 2B satellites (S2A and S2B) have been deployed by the European Space Agency (ESA). The images offer 13 spectral bands with a spatial resolution between 10 m and 60 m. We only keep the 10 bands with a resolution of 10 m and 20 m. The interval between two subsequent acquisitions is 5 days considering both satellites. We use the Sentinel-2 (S2) level-2 images provided by THEIA (download site: https://theia.cnes.fr). These images are corrected for atmospheric effects (*Top-Of-*Canopy, TOC) and have a cloud mask calculated by the MAJA algorithm [3]. The first image of the Sentinel-2B satellite was acquired on Reunion Island on July 6, 2017. The satellite was put into operational service in August 2017. So from August we have an S-2 image every 5 days.

The Landsat-8 (L8) satellite was deployed by NASA (download site: https://earthexplorer.usgs.gov). The revisiting frequency is 16 days. L8 images have a spatial resolution of 15 m for the panchromatic band and 30 m for the multispectral bands. We use TOA level radiometrically corrected images, and pre-process the whole reflectance stack to a 15 m spatial resolution using pansharpening.

The characteristics of the L8 and S2 images are different, but in tropical areas with high cloud cover, the combination of these sensors increases the probability of regularly observing the entire territory.

**Fig. 5:**
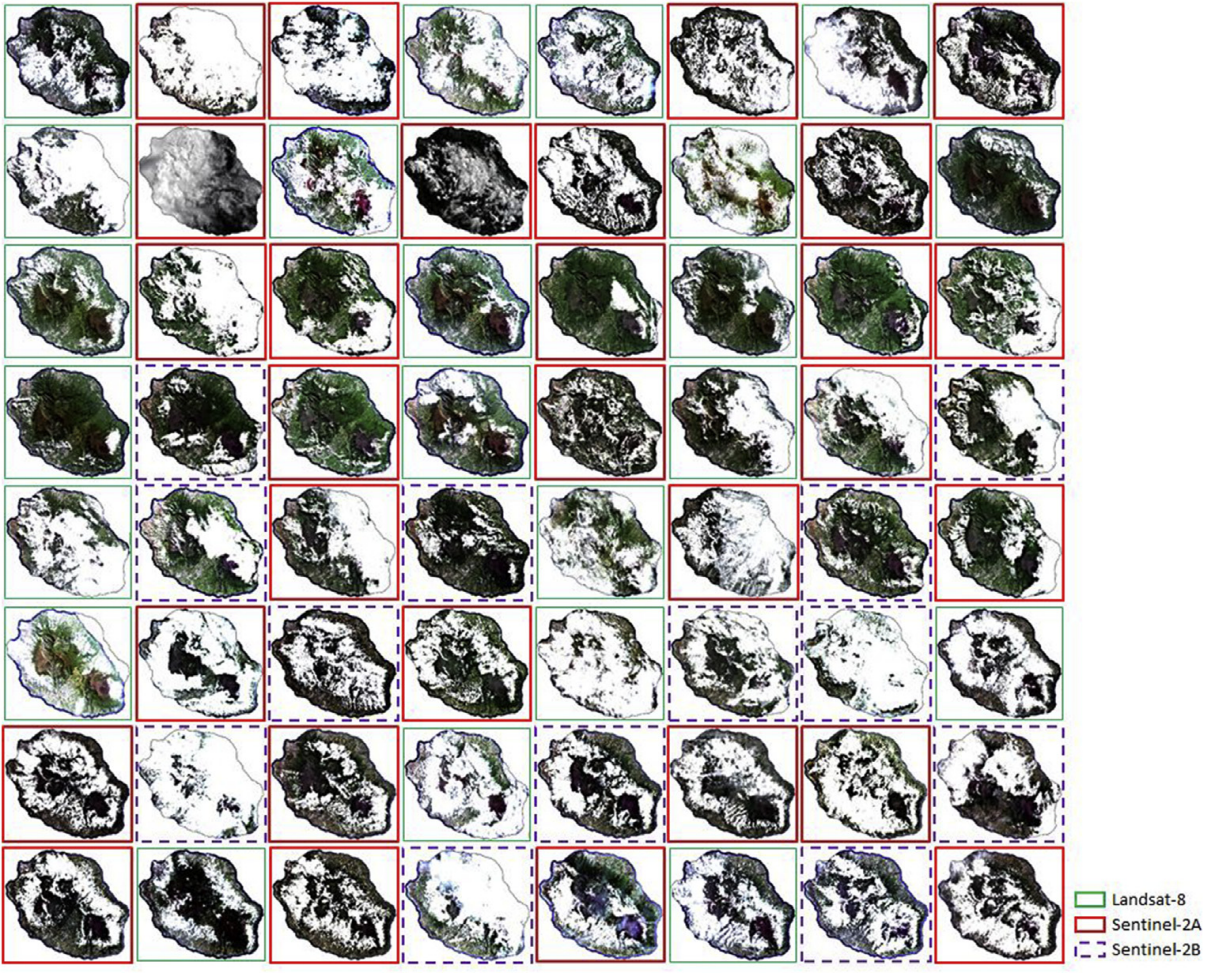
Thumbnails of the 64 images in the time series used to produce the 2017 land cover map with a distinction of the sensors used: Sentinel-2A, Sentinel-2B and Landsat-8.

The time series consists of 64 images acquired between January 1 and December 31, 2017. That is 22 L8, 29 S2A and 13 S2B. The selection criteria for these images are: cover at least 20% of the study area and have less than 80% cloud cover per tile. Fig. 5 illustrates the finally selected images.

#### 2.1.3 Topography

The Digital Terrain Model (DTM) used is the IGN's “RGE ALTI®” product with a resolution of 5 m. This product is obtained by combining several types of acquisition (LiDAR, radar and aerial photography). The production method is described in this document: (http://professionnels.ign.fr/doc/DC_RGEALTI_2-0.pdf).

### 2.2 The Moringa processing chain

The TETIS laboratory is developing an automated soil classification method based on the Moringa chain that minimizes interactions with users by automating most image analysis and processing processes [4]. The Moringa chain can be downloaded with this link: https://gitlab.irstea.fr/raffaele.gaetano/moringa.

**Fig. 6:**
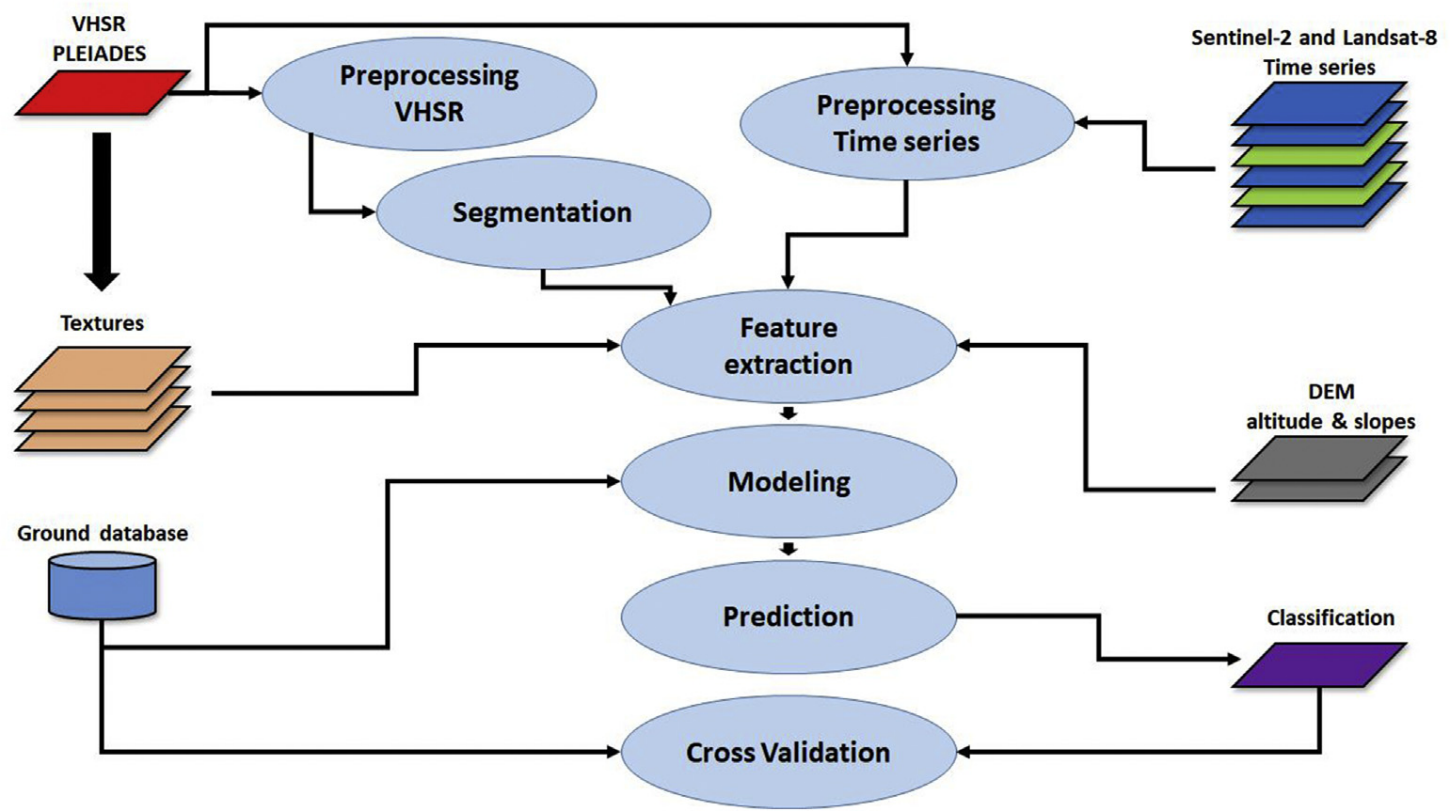
The moringa workflow.

The methodology jointly uses a Spot6/7 or Pleiades Very High Spatial Resolution (VHSR) image, time series of Sentinel-2 and Landsat-8 High Spatial Resolution (HRS) optical images and a Digital Terrain Model (DTM) for Object Based Image Analysis (OBIA) classification (use of the Random Forest algorithm) driven by a learning database combining in situ and photo-interpretation measurements. The chain is built upon the Orfeo Tool Box (OTB) applications, orchestrated by python scripts. Some pre-processing steps are performed under QGiS. The main processes used by the chain are summarized in Fig. 6. In the following paragraphs, we will describe the elements that present specific parameters adapted to the production of the maps presented here and other details useful for a full understanding of the method used.

#### 2.2.1 VHSR pre-processing

**Fig. 7:**
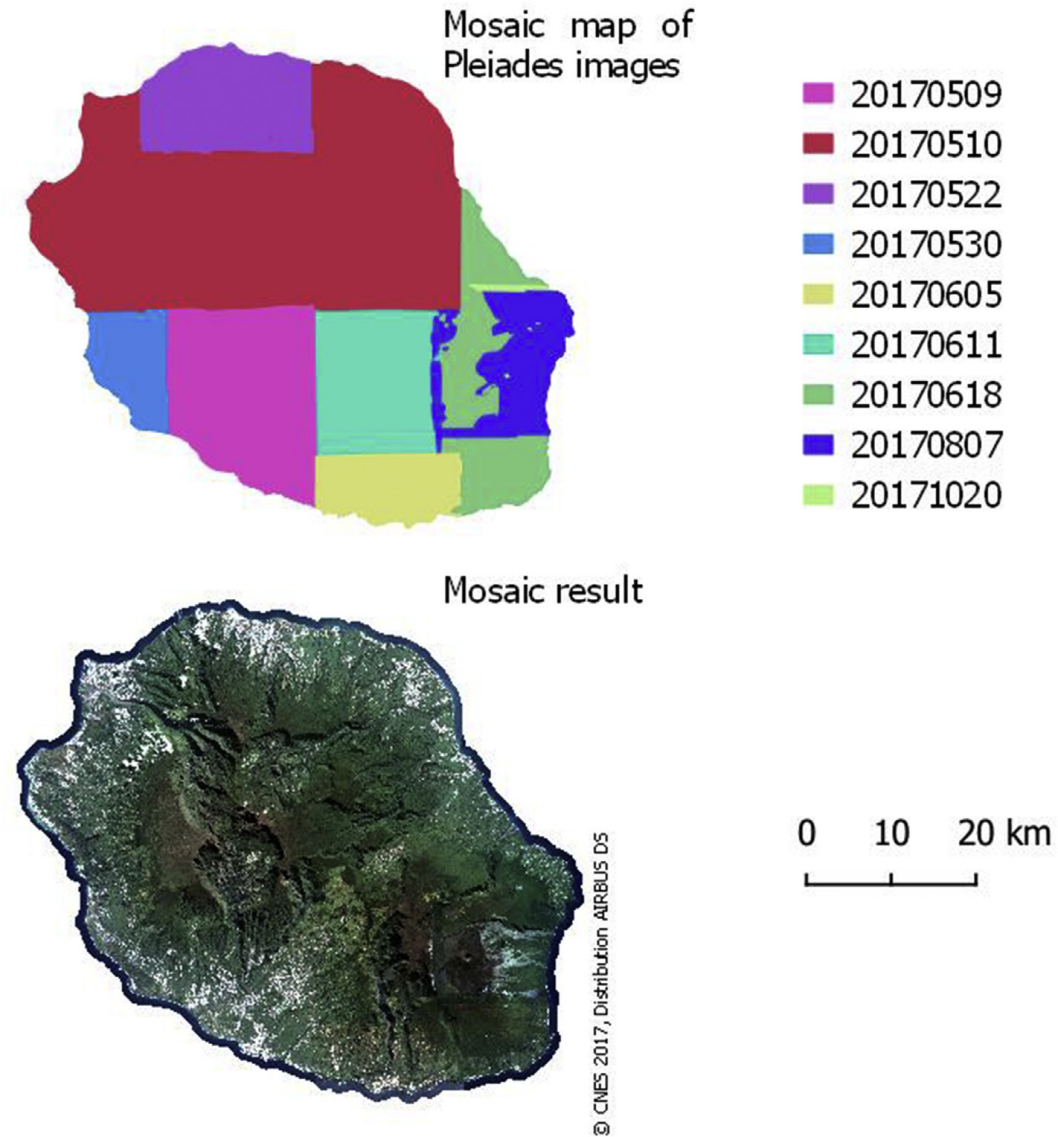
; Mosaic composition map and final harmonized Pléiades mosaic for 2017.

To combine the Pleiades images, we used the OTB mosaic tool described in the article [5] which allows radiometric equalization between the images. The assembly map and the image produced are illustrated in Fig. 7 which shows the areas covered by each image in the mosaic.

#### 2.2.2 HSR pre-processing

Pre-processing applied to HRS images is automated in the Moringa chain. For S2, all bands are resampled at 10 m. For L8, pansharpening processing is applied to bring the spatial resolution as close as possible to S2 images and a cloud cover mask is calculated with the F-Mask tool [6].

An automatic co-registration onto the THRS image is performed automatically by a procedure based on homologous point extraction. This processing is conceived to improve overlapping among the different remote sensing dataset, and is mostly important for the characterization of smaller scale objects.

**Fig. 8:**
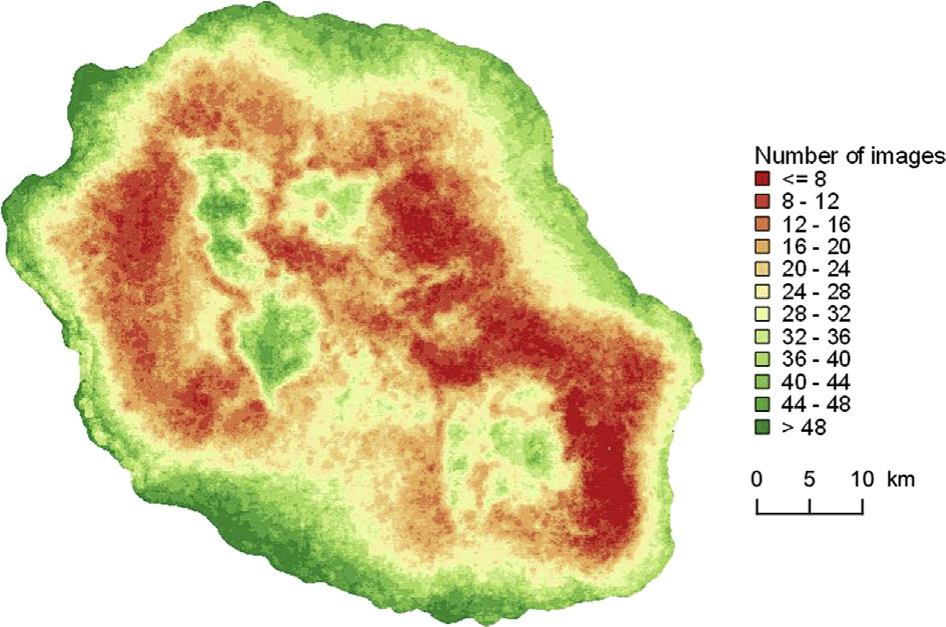
Number of images without clouds in 2017 time serie (include Sentinel 2 and Landsat 8).

Cloud and shadow masks are then used to fill gaps due to cloud coverage into the time series by means of a multi-temporal interpolation [7]. The chain produces, from the cloud masks, an image illustrating the number of times a pixel is not covered by clouds in the time series. This illustration locates the areas where there is a risk of instability of the results on the maps if the number of images is low (Cf. Fig. 8).

#### 2.2.3 The variables used in the classification

➢ We have selected the indices that we believe are most useful for extracting our classes of interest (Cf. Table 2)
➢ Textures are important to detect patterns visible on the THRS image such as tree alignments in tree crops. In the Moringa chain, these texture indices are the only variables derived from the VHSR image. We use the OTB “Haralick Texture Extraction” algorithm applied to the panchromatic image (Cf. Table 2)
➢ Slopes were also calculated on this data using QGIS software. DTM and slopes are used as variables in the classification process.

#### 2.2.4 Object based classification

We use the Random Forest classification algorithm [14] which is adapted for heterogeneous data. This is our case since we use data from several sensors but also altitude, slopes and texture indices. RF is in our case applied to objects extracted in the image via a segmentation step. This step allows to extract objects corresponding to homogeneous groups of pixels in the THRS image. We use the method described in Ref. [15] with OTB's GenericRegionMerging (Large Scale version) application [16]. To obtain a segmentation result adapted to our study, we act on both the homogeneity criteria and the maximum heterogeneity threshold. We have chosen the following parameters:

➢ Scale parameter: 150
➢ Weight parameter on the shape: 0.1
➢ Weight parameter on compactness: 0.5

To apply RF to segmentation objects, zonal statistics are extracted for each object using the average of the pixels of each variable from the time series, textures, slopes and altitude (Cf. Table 2). We perform an independent training and classification for each level of nomenclature.

**Table 2:** Description of variables extracted to compute the classification (with HSR = High Spatial Resolution and VHSR = Very High Spatial Resolution).

Type	HSR	VHRS	Topography
Spectral indices	NDVI^1^ [8], MNDVI^2^ [9], NDWI^3^ [10], MNDWI^4^ [11], brightness index^5^ and RNDVI^6^ [12]		
Textural indices		Energy, Contrast Correlation et Variance Haralick indices [13] calculated at 2 windows size: 11 × 11 and 43 × 43	
Topographic indices			Altitude and slope
**number**	**362**	**5**	**2**

1: Normalized Difference Vegetation Index. 2: Modified Normalized Difference Vegetation Index. 3: Normalized Difference Water Index. 4: Modified Normalized Difference Water Index. 5: Square root of the sum of squared values of all bands. 6: Rededge NDVI (only for Sentinel-2).

#### 2.2.5 Validation

We here use the k-fold cross-validation technique to evaluate the accuracy of the provided land use maps. We decide to avoid using the classical hold out strategy (i.e., leaving a single subset aside for validation) because of the strong intra-class variability which can bias the evaluation. After dividing the field database into approximately equal k subsets (with k = 5 in our case), a cross-validation is performed by training five Random Forest models with different combination of four out of five subsets and validating with using the fifth one, so that each subset is used once for validation and 4 times for training.

The final precision metrics (global accuracy, Kappa, fscore, etc.) are obtained by averaging the metrics obtained at each turn (fold) of the cross-validation. This process is performed for each level of the nomenclature.

➢ Overall accuracy: refers to the portion of well-classified area in relation to the total annotated surface. It does not provide information on the most reliable classes.
➢ The Kappa index (or KIA) takes into account the errors in the rows and columns of the confusion matrix. It expresses the reduction in error compared to that obtained by a classification that would be carried out randomly. It varies from 0 to 1. For example, a value of 0.75 expresses that the classification method used avoids 75% of the errors obtained by a procedure working completely at random.
➢ The F1-score is the harmonic mean of the user's and producer's accuracies given by the confusion matrix.

These quality indicators are given in Table 3.

**Table 3:** Global and class accuracy indices by level.

Level 1	F1-Score	Level 2	F1-Score	Level 3	F1-Score
Agricultural area	94.98%	Sugar cane	94.44%	Sugar cane	90.31%
				Replanted sugar cane	73.21%
		Pasture and feed	93.51%	Pasture	75.76%
				Feed	78.86%
		Gardening	68.30%	Other gardening crop	42.69%
				Potato	12.74%
				Pineapple	69.28%
				Culture on trellis (pitaya, passion fruit, vine)	0%
		Greenhouse	55.62%	Greenhouse	59.34%
		Fruit crop	81.91%	Citrus orchard	50.97%
				Litchi or longane orchard	79.62%
				Mango	70.83%
				Coconut groves	47.33%
				Banana plantation	60.75%
Natural space and forest plantation	97.98%	Woodland	91.29%	Mountains rainforest	85.25%
				semi-dry forest	12.80%
				Other tree vegetation	73.88%
				Forest plantation	90.59%
		Shrub and herbaceous savannah	86.98%	High-altitude scrubland	87.93%
				Scrubland on cliff	79.92%
				Herbaceous vegetation in high altitude	62.97%
				Herbaceous savannah in low altitude	83.37%
				Rubus alceifolius areas	87.17%
				Natural vegetation on lava flow	87.25%
		Rock and natural bare soil	96.21%	Recent volcanic rock	97.76%
				Rock and natural bare soil	79.89%
				Beach and coastal rock	31.37%
		Shadow due to relief	95.91%	Shadow due to relief	95.80%
Water	96.42%	Water	98.67%	wetland	95.65%
				Water	99.47%
				Hill reservoirs	0%
Impervious area	86.83%	Impervious area	85.80%	Built-up area	78.30%
				Photovoltaic panel	96.70%
				Roads and parking	69.65%
Overall accuracy	96.96%		91.88%		86.20%
Kappa index	0.9409		0.9042		0.8486

### 2.3 Post classification

➢ Smoothing by majority filter:

We apply a majority filter to the classification to smooth out contours and remove isolated pixels. We use OTB's Classification Map Regularization tool. The size of the structuring element can be adjusted to measure the intensity of the smoothing. To limit the degradation of the classification, a filter of radius 1, corresponding to a 3 × 3 pixel window, is chosen.

➢ Crossing with GIS layers:

To make corrections to the classification results we use the SAFER SAUP layer which identifies the arable lands. Coupling the output of the classification with this layer makes it possible to switch all polygons detected as cropland outside arable land to natural areas.

## CRediT author statement

Dupuy Stéphane: Conceptualization, Data Curation, Visualization, Investigation, Writing - Original Draft, Gaetano Raffaele: Methodology, Software, Supervision, Writing - Review & Editing, Le Mézo Lionel: Investigation.
